# Dosimetric evaluation of the Acuros XB algorithm for a 4 MV photon beam in head and neck intensity‐modulated radiation therapy

**DOI:** 10.1120/jacmp.v16i4.5222

**Published:** 2015-07-08

**Authors:** Kimiko Hirata, Mitsuhiro Nakamura, Michio Yoshimura, Nobutaka Mukumoto, Manabu Nakata, Hitoshi Ito, Haruo Inokuchi, Yukinori Matsuo, Takashi Mizowaki, Masahiro Hiraoka

**Affiliations:** ^1^ Department of Radiation Oncology and Image‐applied Therapy Graduate School of Medicine, Kyoto University Kyoto Japan; ^2^ Clinical Radiology Service Division Kyoto University Hospital Kyoto Japan

**Keywords:** Acuros XB algorithm, intensity‐modulated radiation therapy, head and neck cancer, low‐energy photon beam, heterogeneous media

## Abstract

In this study, we assessed the differences in the dose distribution of a 4 MV photon beam among different calculation algorithms: the Acuros XB (AXB) algorithm, the analytic anisotropic algorithm (AAA), and the pencil beam convolution (PBC) algorithm (ver. 11.0.31), in phantoms and in clinical intensity‐modulated radiation therapy (IMRT) plans. Homogeneous and heterogeneous, including middle‐, low‐, and high‐density, phantoms were combined to assess the percentage depth dose and lateral dose profiles among AXB, AAA, and PBC. For the phantom containing the low‐density area, AXB was in agreement with measurement within 0.5%, while the greatest differences between the AAA and PBC calculations and measurement were 2.7% and 3.6%, respectively. AXB showed agreement with measurement within 2.5% at the high‐density area, while AAA and PBC overestimated the dose by more than 4.5% and 4.0%, respectively. Furthermore, 15 IMRT plans, calculated using AXB, for oropharyngeal, hypopharyngeal, and laryngeal carcinomas were analyzed. The dose prescription was 70 Gy to 50% of the planning target volume (${\text{PVT}}_{70}$). Subsequently, each plan was recalculated using AAA and PBC while maintaining the AXB‐calculated monitor units, leaf motion, and beam arrangement. Additionally, nine hypopharyngeal and laryngeal cancer patients were analyzed in terms of ${\text{PTV}}_{70}$ for cartilaginous structures (${\text{PVT}}_{70\_\text{cartilage}}$). The doses covering 50% to ${\text{PTV}}_{70}$ calculated by AAA and PBC were 2.1%±1.0% and 3.7%±0.8% significantly higher than those using AXB, respectively (p<0.01). The increases in doses to PTV70_cartilage calculated by AAA and PBC relative to AXB were 3.9% and 5.3% on average, respectively, and were relatively greater than those in the entire ${\text{PVT}}_{70}$. AXB was found to be in better agreement with measurement in phantoms in heterogeneous areas for the 4 MV photon beam. Considering AXB as the standard, AAA and PBC overestimated the IMRT dose for head and neck cancer. The dosimetric differences should not be ignored, particularly with cartilaginous structures in PTV.

PACS number: 87.55.‐x, 87.55.dk, 87.55.kd

## I. INTRODUCTION

Dose calculation accuracy is one of the most important steps in the radiation therapy treatment process; however, the dose calculation process is imperfect due to measurement uncertainties, inadequacies in beam modeling, and inherent limitations in the algorithms.

To maximize the therapeutic benefit of radiation therapy, it is essential that the estimated doses to tissues are delivered accurately. The human body consists of various tissues and cavities with different physical and radiological properties. Previous‐generation dose calculation algorithms, such as the pencil beam convolution (PBC) algorithm, have not accurately calculated the doses for these tissue inhomogeneities. Current‐generation dose calculation algorithms that have been used most commonly in clinical practice, such as the analytic anisotropic algorithm (AAA) and the convolution/superposition algorithm, enable moderate estimates of the changes in dose at locations at which electron disequilibrium exists.^(1)^

A newly released dose calculation algorithm was recently developed called the Acuros XB (AXB) algorithm, which explicitly solves the Linear Boltzmann Transport Equation (LBTE). ^(2)^ LBTE is the governing equation that describes the distribution of radiation particles resulting from their interactions with matter. AXB directly discretizes the space, angle, and energy variables of the LBTE into grids and calculates the energy fluence variation of electrons and scattered photons in a substance. Some investigations have shown that AXB could achieve comparable accuracy to Monte Carlo methods, which are widely considered the gold standard for accurate dose calculation used in radiation therapy in phantom experiments, assuming the presence of homogeneous water and heterogeneous media.^3^, ^4^

Widely differing densities are evident among the head and neck region (soft tissue, bone, air cavities); moreover, primary tumors often exist next to air in the oral or pharyngeal cavities and in some cartilaginous structures. Additionally, certain treated nodal regions are located at the shallow subcutaneous area in head and neck cancer. As reported by Petoukhova et al.,^(5)^ the 4 MV photon beam produced a somewhat smaller underdosage effect behind air cavities and a faster rebuildup than the 6 MV photon beam in experiments using the larynx phantom. Therefore, the 4 MV photon beam could be advantageous over the 6 MV beam for avoiding underdosage to treatment targets located at the air/tissue interface. However, no study has reported on AXB using the 4 MV photon beam.

Additionally, the target volumes are surrounded by several organs at risk (OARs) in the head and neck region; therefore, long‐term toxicities of radiation therapy are highly prevalent with three‐dimensional conformal radiation therapy. Particularly, radiation‐induced xerostomia is one of the most commonly reported long‐term side effects of radiation therapy for head and neck cancers. Intensity‐modulated radiation therapy (IMRT) can spare the major salivary glands and aid saliva flow recovery.^(6)^ Kan et al.^(7)^ assessed the dosimetric impact of using AXB instead of AAA for the treatment of nasopharyngeal carcinoma with IMRT using a 6 MV photon beam. However, no report has evaluated the dosimetric performance of AXB for oropharyngeal, hypopharyngeal or laryngeal carcinoma treated with IMRT. Kan and colleagues also stratified the target volumes into three groups — tissue, air, and bone targets — and evaluated the dose distributions to each group. In addition to this classification, assessing the dosimetric impact on pharyngeal cartilages should also be meaningful, because the cartilaginous structures are heterogeneous components of the target volume and clinically important organs that can be invaded by progressive primary tumors for hypopharyngeal and laryngeal carcinoma.^(8)^

The purpose of this study consisted of two phases. First, we assessed the differences in dose distribution of a 4 MV photon beam among different calculation algorithms, including the PBC, AAA, and AXB, using homogeneous and heterogeneous phantoms. Second, to assess the dose distributions and dose‐volumetric data, IMRT plans for oropharyngeal, hypopharyngeal, and laryngeal carcinoma patients using AXB were recalculated for PBC and AAA while maintaining the AXB‐calculated monitor units and beam arrangement.

## II. MATERIALS AND METHODS

### A. Phantom study

We investigated the differences in dosimetric performance among AXB, AAA, and PBC using homogeneous water phantom and heterogeneous slab phantom for measurement. In homogeneous water phantom, the assigned computed tomography (CT) value was 0 Hounsfield unit (HU) with a physical density (ρ) of $1.0000\;g/{\text{cm}}^{3}$ and electron density relative to water ($\rho^{*}$) of 1.0000, corresponding to “water” as an assigned material. In heterogeneous phantom, the assigned CT values were averaged HU values for CT images obtained by scanning the respective phantoms. In order to verify the calculated dose distribution under a condition close to patient calculation, physical density and corresponding material were automatically assigned to the heterogeneous slabs in this study. The material composition of a given voxel in a 3D CT image was assigned automatically from the CT value based on our own CT calibration curve determined from the Gammex 467 Tissue Characterization Phantom (Gammex, Inc., Middleton, WI) and the material data in AXB (ver. 11) (Acuros; Varian Medical Systems, Palo Alto, CA). The automatic assignment of the materials in AXB includes human material such as “Air”, “Lung”, “Adipose Tissue”, “Muscle Skeletal”, “Cartilage”, and “Bone”. According to range of CT values, there are overlapping physical density regions weighted linearly between two adjacent materials.

**Figure 1:**
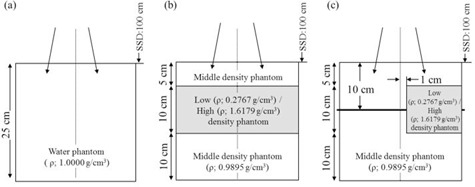
Geometries of the (a) water phantom, (b) phantom with a heterogeneous layer, and (c) phantom with a heterogeneous insert (ρ indicates physical density).

To evaluate the percentage depth dose (PDD) and lateral dose profile, the following three phantoms were combined: 1) a phantom representing a middle‐density area (TM phantom; 20 HU; Taisei Medical, Inc., Osaka, Japan) assigned to the mixed material of “Adipose Tissue” and “Muscle Skeletal” with a physical density (ρ) of $0.9895\;g/{\text{cm}}^{3}$ and electron density relative to water ($\rho^{*}$) of 0.9780; 2) a phantom representing a low‐density area (RMI‐455; 710 HU, Gammex Inc.) assigned to the material of “Lung” with a ρ of $0.2767\;g/{\text{cm}}^{3}$ and $\rho^{*}$ of 0.2729; and 3) a phantom representing a high‐density area (RMI‐450; 920 HU; Gammex, Inc.) assigned to the material of “Bone” with a ρ of $1.6179\;g/{\text{cm}}^{3}$ and $\rho^{*}$ of 1.5196. The phantom was the specific slab with or without a hole hosting an ion chamber (CC04, IBA Dosimetry, Schwarzenbruck, Germany). The geometries of these phantoms are shown in Fig. 1.

The measurements and dose calculations were performed using the CLINAC‐6EX linear accelerator (Varian Medical Systems) with a 4 MV photon beam in the composite phantom at a source‐to‐surface distance of 100 cm with a gantry angle of 0°. Dose calculations were performed using Eclipse version 11.0.3 (Varian Medical Systems). The dose was reported by the dose‐to‐medium ($D_{m}$) mode for AXB. PDD was evaluated using a phantom with a homogeneous and heterogeneous layer in Figs. 1(a) and (b) for a field size of $10\times 10\;{\text{cm}}^{2}$. Depth dose was measured using a small ion chamber with a sensitive volume of $0.04\;{\text{cm}}^{3}$ (CC04; IBA Dosimetry). In order to compare calculations with dose to medium measurements, the stopping power ratio with the correct materials of 1.011 and 0.965 were used for the low‐ and high‐density area, determined by reference to the publication by Araki.^(9)^ We used specific slab phantoms with or without a hole hosting the ion chamber. The PDD curve was then generated by normalizing depth dose at maximum dose depth of 1.0 cm. The lateral dose profile was obtained using a phantom with the low‐ or high‐density insert in Fig. 1(c) with a field size of $10\times 10\;{\text{cm}}^{2}$ and was normalized to the dose on the beam central axis at a depth of 10 cm. The lateral dose profile was measured using EDR2 film (Kodak, Rochester, NY).

### B. Planning study

#### B.1 Patient selection and volume definition of targets and organs at risk

The treatment plans for 15 patients who underwent simultaneous integrated boost IMRT (SIB‐IMRT) to treat oropharyngeal, hypopharyngeal, and laryngeal carcinoma in our institution between March and October 2013 were enrolled in the present study.

For the treatment planning, CT was performed using 100 cc iodinated contrast agent at a flow rate of 1 cc/sec. The scan was initiated 120 s after the start of the injection. CT was performed with the immobilization device in the treatment position and a slice thickness of 2.5 mm. The primary gross tumor volume (pGTV) and nodal gross tumor volume (nGTV) were contoured based on all available imaging methods, such as contrast‐enhanced CT, magnetic resonance imaging, and F18‐fluorodeoxyglucose positron emission tomography, as well as clinical examination. A clinical target volume (${\text{CTV}}_{70}$) was created by adding a 5 mm margin to each GTV. The high‐risk subclinical target volume (${\text{CTV}}_{63}$) was determined by adding 5–20 mm margins to the GTV, depending on tumor location and disease stage. Then, anatomical boundaries based on the microscopic invasion were edited manually. The ${\text{CTV}}_{63}$ also included both node‐positive areas and adjacent nodal regions. The ${\text{CTV}}_{56}$ was defined as the remaining neck nodal areas considered low risk for potential microscopic spread. The planning target volumes prescribed for 70, 63, and 56 Gy (${\text{PTV}}_{70}$, ${\text{PTV}}_{63}$, and ${\text{PTV}}_{56}$, respectively) were created by adding a 5 mm margin to the ${\text{CTV}}_{70},{\text{CTV}}_{63}$, and ${\text{CTV}}_{56}$. PTVs were restricted to skin cropping at 2 mm from the surface. Surrounding critical normal structures, including the brainstem, spinal cord, parotid glands, mandible, and larynx, were contoured. The planning organs‐at‐risk volumes (PRVs) of the brainstem and spinal cord were outlined using a 5 mm margin for each organ (PRVStem, PRVCord).

#### B.2 Treatment planning

IMRT plans were created using a seven‐field sliding window technique via Eclipse and the Dose Volume Optimizer version 11.0.3 (Varian Medical Systems). To avoid uncertainties in oral reproducibility and metal artifacts in teeth, we used typical gantry angles of 80°, 120°, 150°, 180°, 210°, 240°, and 280°. All treatment plans were generated using 4 MV photon beams from CLINAC‐6EX with a Millennium 120 multileaf collimator.

The dose prescription was 70 Gy in 35 fractions (2 Gy per fraction) to 50% of the ${\text{PVT}}_{70}$. The clinical objectives for the optimization are shown in Table 1. Dose calculations were performed using AXB version 11.0.31 with a heterogeneity correction and 2.5 mm grid resolution. Dose calculations were made in the $D_{m}$ mode. The material substances were allocated automatically using the predetermined material table (ver. 11.0). Subsequently, each plan was recalculated using AAA and PBC version 11.0.31 with heterogeneity correction and the same monitor units and leaf motions as the plan using AXB.

**Table 1:** Clinical objectives for the optimization

Structure	Index	Per Protocol	Acceptable Variation
${\text{PVT}}_{70}$	$D_{50}$	=100%	98%–103%
	$D_{98}$	>93%	>90%
	$D_{2}$	<105%	<115%
${\text{PTV}}_{63}$ ^*^	$D_{90}$	=100%	>97%
	$D_{50}$	<105%	<108%
${\text{PTV}}_{56}$ ^†^	$D_{90}$	=100%	>97%
	$D_{50}$	<105%	<108%
${\text{CTV}}_{70}$	$D_{95}$	>100%	>98%
${\text{CTV}}_{63}$	$D_{95}$	>100%	>98%
${\text{CTV}}_{56}$	$D_{95}$	>100%	>98%
GTV	$D_{95}$	>100%	>98%
PRV_Stem	$D_{2\text{cc}}$	<54 Gy	<60 Gy
Brainstem	Maximum dose	<54 Gy	<60 Gy
PRV_Cord	$D_{2\text{cc}}$	<45 Gy	<50 Gy
Spinal cord	Maximum dose	<45 Gy	<50 Gy
Lt. Parotid	$V_{30\text{Gy}}$	<50%	
Rt. Parotid	$V_{30\text{Gy}}$	<50%	

a ^*^ The ${\text{PTV}}_{63}$ index percentages are calculated based on a prescribed dose of 63 Gy.

b ^†^ The ${\text{PTV}}_{56}$ index percentages are calculated based on a prescribed dose of 56 Gy.

D=the dose covering X% volumes; $D_{2\text{cc}}=\text{the dose received in a volume of at least}\;2\;\text{cc}$; $V_{30\text{yG}}=\text{volume receiving}\;30\;\text{Gy}$; Lt.=left; Rt.=right.

#### B.3 Dosimetric evaluation

The following dose‐volumetric data calculated using AXB, AAA, and PBC were compared with the PTVs: the doses covering 50% volumes ($D_{50}$), minimum doses represented by doses covering 98% volumes ($D_{98}$), and maximum doses represented by doses covering at least 2% volumes ($D_{2}$). The homogeneity index (HI) for the ${\text{PTV}}_{70}$ was calculated as the ratio $(D_{2}-D_{98})/D_{50}$ to evaluate target dose homogeneity.

**Figure 2:**
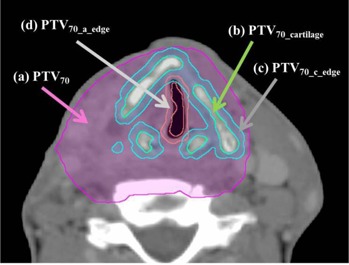
An example of the axial image of a hypopharyngeal cancer patient indicating the structure of (a) ${\text{PTV}}_{70}$, (b) PTV70_cartilage, (c) ${\text{PTV}}_{70\_c\_\text{edge}}$, and (d) ${\text{PTV}}_{70\_a\_\text{edge}}$.

In addition, nine hypopharyngeal and laryngeal cancer patients were analyzed in terms of the ${\text{PTV}}_{70}$ for the hyoid bone, thyroid, and cricoid cartilages (${\text{PTV}}_{70\_\text{cartilage}}$), which are heterogeneous components of the ${\text{PTV}}_{70}$ and clinically important organs that can be invaded by progressed primary tumors.^(8)^ Cartilaginous structures in ${\text{PTV}}_{70}$ were first delineated by autosegmentation using the CT ranger tool with range of 120–3000 HU, with processing in 3D mode. The corresponding materials were “Cartilage” and “Bone” for 120 HU, and “Bone” for 3000 HU. Thus, there were overlapping physical density regions for two adjacent materials. Then, the contours were modified manually according to the patient's anatomy by a radiation oncologist. The means and standard deviations (SDs) of CT values were measured to evaluate variations in contouring cartilaginous structures. We also created ${\text{PTV}}_{70\_c\_\text{edge}}$ and ${\text{PTV}}_{70\_c\_\text{edge}}$ by generating a shell via a 2 mm expansion of the cartilaginous structures and air cavity, respectively, using the autosegmentation tool, to evaluate the dose near the cartilage/tissue and air/tissue interfaces, respectively. Examples of the structures are shown in Fig. 2.

Clinically relevant dose‐volumetric data for the OARs were compared. The doses received in volumes of at least 2 cc ($D_{2\text{cc}}$) to the brainstem and spinal cord were reported. Those volumes receiving 30 Gy ($V_{30\text{Gy}}$) and the mean doses to the parotid glands were reported, as were the mean dose differences to the larynx in oropharyngeal cancer patients.

To determine the statistical significance of the observed differences, two‐sided paired *t*‐tests were used. A p‐value less than 0.05 was deemed to indicate statistical significance.

## III. RESULTS

### A. Phantom study

**Figure 3:**
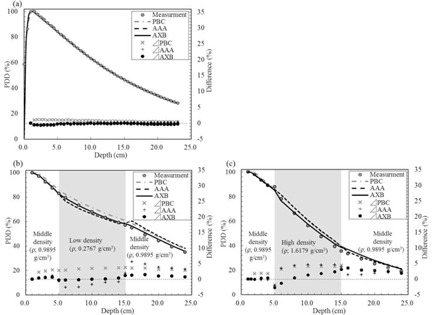
PDD of 4 MV photon beams in the (a) water phantom, (b) low‐density phantom, and (c) high‐density phantom. Differences are represented as PDD (calculation) ‐ PDD (measurement).

PDD values on the homogenous and heterogeneous phantoms are shown in Fig. 3. For the homogeneous phantom, each algorithm was in agreement with measurement within 1.5%, particularly AXB and AAA, which were within 1.0%. Additionally, regarding the phantom containing the low‐density area, the dose calculated using AXB showed agreement with measurement within 0.5% at the low‐density area and within 1.5% at the rebuildup area. The greatest differences between AAA and PBC calculations and measurement were 2.7% and 3.6%, respectively, in the low‐density area. AAA and PBC overestimated the dose distribution by more than 5.5% and 3.5%, respectively, at the rebuildup area. For the phantom containing the high‐density area, the dose calculated using AXB was in agreement with measurement within 2.5% at the high‐density area, but was overestimated at the high‐density/tissue interface, with a difference of 3.5%. AAA and PBC overestimated the dose distribution in the high‐density area by more than 4.5% and 4.0%, respectively.

**Figure 4:**
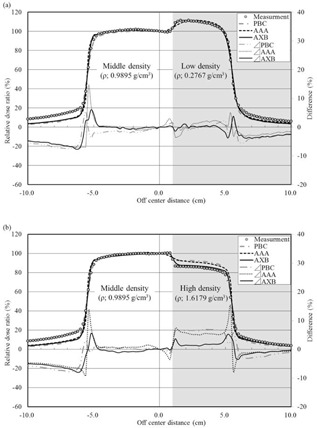
Lateral dose profile of 4 MV photon beams in the (a) low‐density phantom and (b) high‐density phantom. Differences are represented as PDD (calculation) ‐ PDD (measurement).

Lateral dose profiles are shown in Fig. 4. With the low‐density phantom, the greatest differences obtained near the boundary (off‐center distance of x=0.8 cm) were 0.4%, 2.9%, and 1.2% for AXB, AAA, and PBC, respectively. Additionally, the greatest differences obtained near the boundary (off‐center distance of x=0.8 cm) were 0.3%, 3.4%, and 1.3% for AXB, AAA, and PBC, respectively, in the high‐density phantom. The averaged doses in the high‐density area (from a 2.0 to 4.0 cm off‐center distance) calculated by AXB, AAA, and PBC were 1.5%, 5.3%, and 6.7% greater than the measurement dose, respectively.

### B. Planning study

All of the treatment plans calculated by AXB were per protocol or at least acceptable for the dose‐volume constraints of our protocol. The dose per fraction to the ${\text{PTV}}_{70}$ was 2 Gy. The averaged total number of monitor units was 1269±137.1 (range, 1037–1534) per fraction.

Table 2 summarizes the averaged dose‐volumetric data of the PTVs for the 15 patients. The $D_{50}$ to ${\text{PTV}}_{70}$ calculated using AAA and PBC were 2.1%±1.0% and 3.7%±0.8% higher, respectively, than those using AXB significantly (p<0.01). The $D_{50}$ to ${\text{PTV}}_{63}$ calculated using AAA and PBC were 1.8%±1.1% and 3.4%±0.6% significantly higher, respectively, than those using AXB (p<0.01). The $D_{98}$ to ${\text{PTV}}_{63}$ estimated using AAA was also significantly higher than that using AXB, whereas no significant difference was found in the $D_{98}$ to ${\text{PTV}}_{63}$ between PBC and AXB estimates.

**Figure 5:**
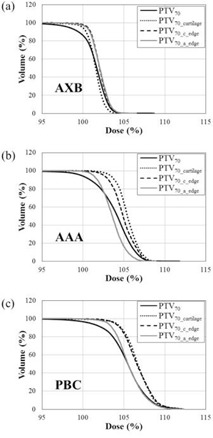
Averaged dose‐volumetric histograms of nine patients calculated using the (a) Acuros XB (AXB) algorithm, (b) analytic anisotropic algorithm (AAA), and (c) pencil beam convolution (PBC) algorithm. The graphs indicate ${\text{PTV}}_{70}$, PTV70_cartilage, ${\text{PTV}}_{70\_c\_\text{edge}}$, and ${\text{PTV}}_{70\_a\_\text{edge}}$.

**Figure 6:**
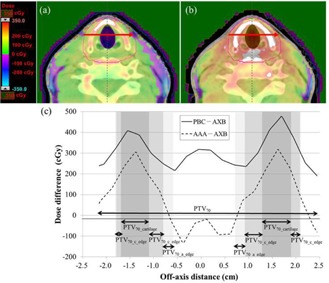
Representative dose differences among AXB, AAA, and PBC: (a) AAA ‐ AXB, (b) PBC ‐ AXB. Upper and lower dose limits show 5% of the prescription dose. Line profiles (c) are indicated by the red arrow in (a) and (b). The gray area in (c) indicates the range of PTV70_cartilage, ${\text{PTV}}_{70\_c\_\text{edge}}$, and ${\text{PTV}}_{70\_a\_\text{edge}}$.

The mean ± SDs of CT value in cartilaginous structures ranged from 179±124 to 319±209 HU (corresponding ρ ranged from 1.1269 to $1.2199\;g/{\text{cm}}^{3}$). The SDs ranged from 124 to 209 HU. The minimum and maximum of mean ± SDs of CT value was 55 and 528 HU, respectively (corresponding ρ ranged from 1.0724 to $1.3736\;g/{\text{cm}}^{3}$). The corresponding materials were “Muscle Skeletal” and “Cartilage” for 55 HU, and “Cartilage” and “Bone” for 528 HU. The averaged dose‐volumetric data of cartilaginous structures and air in ${\text{PTV}}_{70}$ (PTV70_cartilage, ${\text{PTV}}_{70\_c\_\text{edge}}$, and ${\text{PTV}}_{70\_a\_\text{edge}}$) for nine patients with hypopharyngeal and laryngeal carcinoma are shown in Table 3. The $D_{50}$ calculated by AAA was 3.9% higher for PTV70_cartilage than that by AXB, and the $D_{50}$ calculated by PBC was 5.3% higher than that by AXB. The dose increase to PTV70_cartilage calculated by AAA and PBC was greater than that in the entire ${\text{PVT}}_{70}$. For the ${\text{PTV}}_{70\_c\_\text{edge}}$, the $D_{50}$ values calculated by AAA and PBC were higher than those by AXB, and the differences were somewhat greater than the difference in the entire ${\text{PVT}}_{70}$. For the ${\text{PTV}}_{70\_a\_\text{edge}}$, the difference between AAA and AXB was smaller than that in the ${\text{PVT}}_{70}$. Differences in PBC from AXB showed a similar trend. The averaged dose‐volumetric histograms of nine patients for the ${\text{PTV}}_{70}$, ${\text{PTV}}_{70\_\text{caritlage}}$, ${\text{PTV}}_{70\_c\_\text{edge}}$, and ${\text{PTV}}_{70\_a\_\text{edge}}$, calculated using the three algorithms, are shown in Fig. 5. The dose differences between ${\text{PTV}}_{70}$ and ${\text{PTV}}_{70\_\text{caritlage}}$ or ${\text{PTV}}_{70\_c\_\text{edge}}$ were emphasized in AAA and PBC. Figure 6 shows the dose difference distributions between (a) AXB and AAA and (b) AXB and PBC of the axial slice of the thyroid cartilage level in a representative case. Their line profiles are also shown in Fig. 6(c). In particular, AAA and PBC overestimated the dose to the thyroid cartilage, compared with AXB.

Table 4 summarizes the averaged dose‐volumetric data to OARs. Using AAA and PBC, the averaged differences of $D_{2\text{cc}}$ for PRV_Stem were 1.3 Gy and 1.6 Gy higher and those for PRV_Cord were 1.8 Gy and 2.6 Gy higher, respectively, than that using AXB. Little increase was found in the mean dose to the parotid area and volume of $V_{30\text{Gy}}$ estimated using AAA and PBC compared with AXB.

**Table 2:** Averaged dose‐volumetric data for PTVs

*Structure*	*Index*	*AXB*	*AAA*	*PBC*	*Difference AAA‐AXB*	*P*	*Difference PBC‐AXB*	*P*
${\text{PVT}}_{70}$	$D_{50}$ (%)	101.7±0.4	103.8±1.1	105.4±0.9	2.1±1.0	<0.01	3.7±0.8	<0.01
	$D_{98}$ (%)	95.5±2.1	96.8±2.6	97.6±2.6	1.2±1.3	<0.01	2.1±1.2	<0.01
	$D_{2}$ (%)	104.2±1.0	106.9±1.2	109.2±1.5	2.7±1.0	<0.01	5.0±1.3	<0.01
	HI	0.085±0.028	0.098±0.030	0.110±0.030	0.012±0.010	<0.01	0.024±0.014	<0.01
${\text{PTV}}_{63}$ ^*^	$D_{50}$ (%)	104.3±0.6	106.1±1.0	107.6±1.0	1.8±1.1	<0.01	3.4±0.6	<0.01
	$D_{98}$ (%)	94.9±1.9	95.7±2.2	94.4±1.8	0.8±1.3	0.04	−0.5±1.0	0.06
	$D_{2}$ (%)	110.8±1.6	113.2±1.9	115.4±1.5	2.4±1.0	<0.01	4.5±0.7	<0.01
${\text{PTV}}_{56}$ ^†^	$D_{50}$ (%)	104.5±0.6	106.8±1.5	110.2±1.0	2.4±1.3	<0.01	5.7±1.0	<0.01
	$D_{98}$ (%)	96.2±2.2	97.8±2.8	98.3±3.1	1.6±1.5	<0.01	2.1±1.7	<0.01
	$D_{2}$ (%)	110.8±2.0	113.3±2.9	117.0±2.0	2.5±1.3	<0.01	6.1±1.2	<0.01

a ^*^ The ${\text{PTV}}_{63}$ index percentages are calculated based on a prescribed dose of 63 Gy.

b ^†^ The ${\text{PTV}}_{56}$ index percentages are calculated based on a prescribed dose of 56 Gy.

$D_{X}=\text{the dose covering X}\%\;\text{volumes}$; HI=homogeneity index.

**Table 3:** Averaged dose‐volumetric data for PTVs among hypopharyngeal and laryngeal cancer patients

*Structure*	*Index*	*AXB*	*AAA*	*PBC*	*Difference AAA‐AXB*	*P*	*Difference PBC‐AXB*	*P*
${\text{PTV}}_{70}$	$D_{50}$ (%)	101.7±0.3	104.3±1.1	105.6±0.9	2.6±1.0	<0.01	4.0±0.9	<0.01
	$D_{98}$ (%)	96.6±1.2	98.4±1.2	99.1±1.5	1.8±1.3	<0.01	2.4±1.1	<0.01
	$D_{2}$ (%)	103.8±0.4	106.9±1.1	109.1±1.4	3.0±1.1	<0.01	5.3±1.5	<0.01
PTV70_cartilage	$D_{50}$ (%)	101.5±0.3	105.5±0.7	106.9±0.7	3.9±0.7	<0.01	5.3±0.8	<0.01
	$D_{98}$ (%)	99.0±1.2	103.0±1.0	102.8±1.7	4.0±1.2	<0.01	3.9±0.8	<0.01
	$D_{2}$ (%)	103.2±0.3	107.5±0.6	109.3±1.4	4.3±0.7	<0.01	6.1±1.5	<0.01
${\text{PTV}}_{70\_c\_\text{edge}}$	$D_{50}$ (%)	102.0±0.4	105.0±1.0	106.8±0.9	2.9±0.9	<0.01	4.8±1.0	<0.01
	$D_{98}$ (%)	100.0±0.7	102.4±1.2	103.0±0.9	2.4±1.0	<0.01	3.0±0.6	<0.01
	$D_{2}$ (%)	103.8±0.3	106.8±1.0	109.3±1.6	3.0±1.1	<0.01	5.5±1.7	<0.01
${\text{PTV}}_{70\_a\_\text{edge}}$	$D_{50}$ (%)	102.1±0.4	103.6±1.0	105.6±0.9	1.5±1.0	<0.01	3.5±0.8	<0.01
	$D_{98}$ (%)	99.7±0.9	101.0±0.5	102.4±1.2	1.3±0.8	<0.01	2.7±0.6	<0.01
	D2 (%)	104.0±0.3	105.9±0.9	109.0±1.2	1.9±0.9	<0.01	5.0±1.2	<0.01

$D_{X}=\text{the dose covering X}\%\;\text{volumes}$.

**Table 4:** Averaged dose‐volumetric data for OARs

*Structure*	*Index*	*AXB*	*AAA*	*PBC*	*Difference AAA‐AXB*	*P*	*Difference PBC‐AXB*	*P*
PRV Stem	$D_{2\text{cc}}$ (Gy)	46.5±5.6	47.8±5.7	48.1±6.0	1.3±0.9	<0.01	1.6±0.5	<0.01
PRV Cord	$D_{2\text{cc}}$ (Gy)	42.6±0.9	44.5±1.2	45.2±1.0	1.8±0.9	<0.01	2.6±0.5	<0.01
Larynx	Mean dose (Gy)	48.3±2.6	49.0±2.8	50.4±2.8	0.7±0.2	<0.01	2.1±0.2	<0.01
Lt. Parotid	$V_{30\text{Gy}}$ (%)	30.5±9.8	31.1±9.8	32.6±10.6	0.5±0.6	<0.01	2.1±1.1	<0.01
	Mean dose (Gy)	28.4±4.5	29.4±4.5	28.9±4.8	1.0±0.6	<0.01	0.6±0.4	<0.01
Rt. Parotid	$V_{30\text{Gy}}$ (%)	38.3±20.6	38.7±20.4	40.4±20.2	0.4±0.9	0.07	2.1±1.0	<0.01
	Mean dose (Gy)	32.8±11.8	33.8±11.7	33.5±12.2	1.0±0.5	<0.01	0.7±0.6	<0.01

$D_{2\text{cc}}=\text{the dose received in a volume of at least}\;2\;\text{cc}$; $V_{30\text{Gy}}=\text{volume receiving}\;30\;\text{Gy}$; Lt.=left; Rt.=right.

## IV. DISCUSSION

Some reports have shown that AXB could achieve comparable accuracy to that using Monte Carlo methods, which have been considered the gold standard for accurate dose calculation.^3^, ^4^ Kroon et al.^(10)^ compared the PDD using AXB for a 6 MV photon beam on a phantom containing the low‐density area and showed that the AXB was acceptably consistent with measurement. In our study, AXB was found to be in better agreement with measurement compared with AAA or PBC in phantoms including low‐ or high‐density areas for a 4 MV photon beam. The high‐density material in the heterogeneous phantom was assigned to “Bone” and not “Cartilage.” However, AXB was in agreement with the measurement, to within 1.0% with the homogenous water phantom ($\rho =1.0000\;g/{\text{cm}}^{3}$), and within 2.5% in a high‐density area of the heterogeneous phantom ($\rho =1.6179\;g/{\text{cm}}^{3}$), and within 3.5% at the interface. From these results, the dosimetric accuracy of AXB in the cartilaginous structures' density range calculated from our own CT calibration curve ($1.072\leq \rho \leq 1.374\;g/{\text{cm}}^{3}$ from our results) can be estimated to be as high as that in high density. Thus, assessment of the dose distributions calculated using AXB even for a 4 MV photon beam is appropriate for clinical planning, except for the high‐density/tissue interface.

Several investigations have been conducted on the clinical impact of AXB for various sites.^7^, ^10^, ^11^ They compared the dose distributions calculated by AXB with those by AAA in IMRT or volumetric‐modulated arc therapy (VMAT) using 6 or 15 MV photon beams for lung cancer or nasopharyngeal carcinoma. Kan et al.^(7)^ reported that the mean and minimum doses to the ${\text{PTV}}_{70}$ calculated by AXB were lower than those by AAA for nasopharyngeal carcinoma. Our results using a 4 MV photon beam were consistent with their results. The findings of the current study indicated that the mean dose to the ${\text{PTV}}_{70}$ was escalated naturally by 2.1%–3.7% if we maintained the original dose prescriptions and dose‐volume constraints in the switch from AAA or PBC to AXB. Although increasing the dose to the PTV may improve the tumor control, the dose increase to pharyngeal mucosa, muscle, and skin inside the PTV may cause unintentional toxicities. For the reasons mentioned above, care should be taken regarding the radiation‐induced toxicity levels after switching the dose calculation algorithm.

Dosimetric evaluation of cartilaginous structures deserves careful attention for hypopharyngeal and laryngeal carcinomas. The dose differences between AAA or PBC and AXB were emphasized in cartilaginous structures of the ${\text{PVT}}_{70}$. Relative to AXB, AAA and PBC overestimated the dose in cartilaginous structures (Fig. 5). When calculating the dose distributions using PBC or AAA, users should pay attention to the decrease in the doses to cartilaginous structures, particularly for T4 of hypopharyngeal tumors and T3 and T4 of laryngeal tumors. Tumors of these stages macroscopically invade thyroid or cricoid cartilage or hyoid bone, and microscopic invasion is considered clinically to spread throughout the entire cartilage. Thus, it is preferable in those patients with advanced stage to ensure the radiation doses to the cartilaginous structures without increasing their risk of developing cartilage necrosis.

Regarding the dosimetric effects on tissues surrounding the cartilaginous structures, the differences between AXB and AAA or PBC were slightly higher than that in the entire ${\text{PVT}}_{70}$. However, our phantom study showed that some dose calculation errors were observed even when using AXB at a high‐density/tissue interface; thus, accurate evaluation of ${\text{PTV}}_{70\_c\_\text{edge}}$ was difficult in the present study.

The entire cartilaginous structure is not mapped to the material of “Cartilage” in AXB. Cartilaginous structures were assigned automatically not only to “Cartilage”, but also “Bone”, “Muscle Skeletal”, and their mixed materials by AXB. The mixed material might make it difficult to clarify the dose calculation accuracy in AXB. However, in the clinical situation, we do not assign the cartilaginous structure to pure “Cartilage” material manually. Even when the tumor invades cartilaginous structures macroscopically and the cartilaginous structures are replaced with tumor, the CT value may still differ from that of “Cartilage.” Moreover, if a tumor invades a cartilaginous structure microscopically, the cartilaginous tissue may not be destroyed and the CT value may be maintained similar to that of “Cartilage.” Because of the complex material components in cartilaginous structures, this research is appropriate for the clinical situation. Furthermore, the variations in cartilaginous structures showed that the segmentation accuracy of cartilaginous structures was sufficient to evaluate dose distributions in the present study.

Regarding the dose effect of the tissue surrounding the air cavity, even though AAA overestimated the dose by more than 5.5% in the rebuildup area in the phantom experiment, the dose difference in the air/tissue surface calculated using AAA and AXB was comparable to that in the entire PTV in the clinical IMRT plan. Two possible explanations exist. First, multifield irradiation might reduce the overestimation of AAA. Kan et al.^(7)^ reported that the difference between the doses to air calculated using AAA and AXB decreased as the number of fields increased. They stated that part of the dose reduction from each single field was compensated by the increase in the out‐of‐field doses from other fields coming from different directions. Second, there were few beams that reached a tumor via air. The anterior part of the laryngeal cavity rarely included the ${\text{PTV}}_{70}$ for early T stage hypopharyngeal carcinoma, because we used gantry angles from only the lateral and dorsal directions to avoid oral reproducibility and metal artifact uncertainties.

In the current study, we created an IMRT plan using AXB and then recalculated the dose distributions using AAA and PBC. When applying a new calculation algorithm, the dose distribution calculated using the new algorithm should be compared with that recalculated using the previous algorithm. Thereafter, a reconsideration of the conventional dose‐volume constraints will be required in inverse planning, such as in IMRT or VMAT planning, to avoid overdosage to the targets. The appropriateness of switching the dose calculation algorithm should be validated carefully from a clinical viewpoint.

## V. CONCLUSIONS

In this study, we demonstrated that AXB showed good agreement in measurements with phantoms containing low‐ and high‐density areas for a 4 MV photon beam. Relative to AXB as the standard, AAA and PBC overestimated the dose in IMRT for oropharyngeal, hypopharyngeal, and laryngeal carcinomas. The differences should not be ignored, particularly with cartilaginous structures in the ${\text{PVT}}_{70}$; thus, radiation oncologists should pay close attention to those cases with invasion to cartilaginous structures.

## ACKNOWLEDGMENTS

This research was supported in part by a Grant‐in‐Aid for Scientific Research from the Ministry of Education, Culture, Sports, Science, and Technology of Japan (Grant 25253078).

## Supporting information

Supplementary Material
