# Supplementary figures and images for: Dosimetric evaluation of the Acuros XB algorithm for a 4 MV photon beam in head and neck intensity‐modulated radiation therapy
Source: J Appl Clin Med Phys. 2015 Jul 8;16(4):52–64. doi: 10.1120/jacmp.v16i4.5222 (PMC5690026; doi:10.1120/jacmp.v16i4.5222)

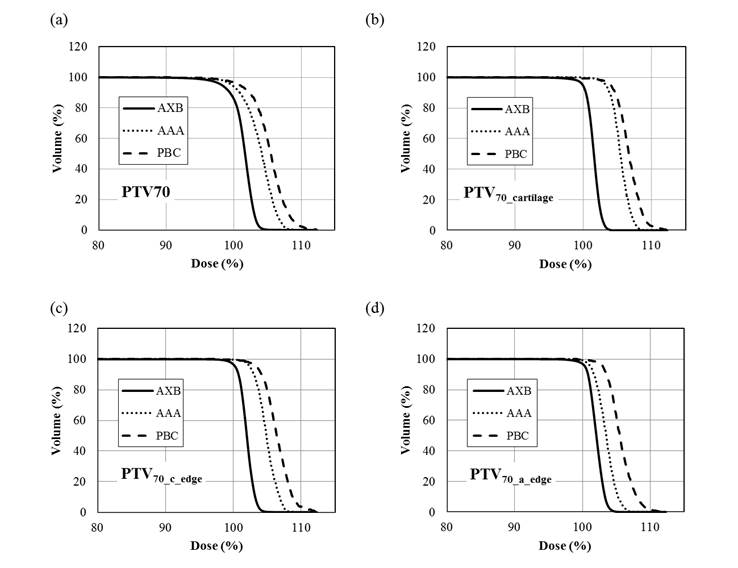

Supplement: Supplementary file 1 — Supplementary Material [file ACM2-16-052-s001.jpg]
